# A multiplexed time-resolved fluorescence resonance energy transfer ultrahigh-throughput screening assay for targeting the SMAD4–SMAD3–DNA complex

**DOI:** 10.1093/jmcb/mjad068

**Published:** 2023-11-15

**Authors:** Wukun Ouyang, Qianjin Li, Qiankun Niu, Min Qui, Haian Fu, Yuhong Du, Xiulei Mo

**Affiliations:** Department of Pharmacology and Chemical Biology, Emory University School of Medicine, Atlanta, GA 30322, USA; Department of Pharmacology and Chemical Biology, Emory University School of Medicine, Atlanta, GA 30322, USA; Department of Pharmacology and Chemical Biology, Emory University School of Medicine, Atlanta, GA 30322, USA; Department of Pharmacology and Chemical Biology, Emory University School of Medicine, Atlanta, GA 30322, USA; Emory Chemical Biology Discovery Center, Emory University School of Medicine, Atlanta, GA 30322, USA; Department of Pharmacology and Chemical Biology, Emory University School of Medicine, Atlanta, GA 30322, USA; Emory Chemical Biology Discovery Center, Emory University School of Medicine, Atlanta, GA 30322, USA; Department of Hematology and Medical Oncology and Winship Cancer Institute, Emory University, Atlanta, GA 30322, USA; Department of Pharmacology and Chemical Biology, Emory University School of Medicine, Atlanta, GA 30322, USA; Emory Chemical Biology Discovery Center, Emory University School of Medicine, Atlanta, GA 30322, USA; Department of Pharmacology and Chemical Biology, Emory University School of Medicine, Atlanta, GA 30322, USA

**Keywords:** TGFβ/SMAD4 signaling, high-throughput screening, TR-FRET

## Abstract

The transforming growth factor-beta (TGFβ) signaling pathway plays crucial roles in the establishment of an immunosuppressive tumor microenvironment, making anti-TGFβ agents a significant area of interest in cancer immunotherapy. However, the clinical translation of current anti-TGFβ agents that target upstream cytokines and receptors remains challenging. Therefore, the development of small-molecule inhibitors specifically targeting SMAD4, the downstream master regulator of the TGFβ pathway, would offer an alternative approach with significant therapeutic potential for anti-TGFβ signaling. In this study, we present the development of a cell lysate-based multiplexed time-resolved fluorescence resonance energy transfer (TR-FRET) assay in an ultrahigh-throughput screening (uHTS) 1536-well plate format. This assay enables simultaneous monitoring of the protein‒protein interaction between SMAD4 and SMAD3, as well as the protein‒DNA interaction between SMADs and their consensus DNA-binding motif. The multiplexed TR-FRET assay exhibits high sensitivity, allowing the dynamic analysis of the SMAD4–SMAD3–DNA complex at single-amino acid resolution. Moreover, the multiplexed uHTS assay demonstrates robustness for screening small-molecule inhibitors. Through a pilot screening of an FDA-approved bioactive compound library, we identified gambogic acid and gambogenic acid as potential hit compounds. These proof-of-concept findings underscore the utility of our optimized multiplexed TR-FRET platform for large-scale screening to discover small-molecule inhibitors that target the SMAD4–SMAD3–DNA complex as novel anti-TGFβ signaling agents.

## Introduction

The remarkable clinical achievements of immune checkpoint inhibitor (ICI) therapy have propelled the rapid advancement of immune-mediated anti-tumor strategies, establishing them as the first-line treatment for various tumor types (Wei et al., 2018; Hahn et al., 2021). Despite the paradigm-shifting progress in cancer immunotherapy over the last decade, the majority of patients fail to respond to current monotherapy based on ICIs, and a significant challenge lies in the occurrence of patient relapse following initial response (Ribas and Wolchok, 2018). Consequently, there is an urgent and unmet clinical need to address the requirements of the majority of cancer patients, necessitating renewed endeavors to broaden the scope and efficacy of immune system-targeted strategies.

Transforming growth factor-beta (TGFβ), signaling has emerged as a promising target for cancer immunotherapy (Colak and Ten Dijke, 2017; Ciardiello et al., 2020). While the role of TGFβ signaling in cancer initiation, progression, and metastasis is multifaceted and context-dependent (Zhao et al., 2018; Batlle and Massague, 2019), its contribution to the establishment of an immunosuppressive tumor microenvironment has been extensively documented for both adaptive and innate immune responses (Zhao et al., 2018; Batlle and Massague, 2019). For instance, TGFβ hampers anti-tumor immunity by inhibiting the proliferation, maturation, differentiation, and activation of natural killer cells, macrophages, dendritic cells, and CD8^+^ T cells (Mariathasan et al., 2018; Batlle and Massague, 2019; Hu et al., 2022). Moreover, it promotes the conversion of naïve CD4^+^ T helper cells into immune suppressive regulatory T cells (Marie et al., 2005). Building upon these observations, anti-TGFβ signaling therapies have been actively investigated in clinical trials, particularly in combination with ICIs, across a wide variety of tumor types.

Numerous therapeutic approaches targeting the anti-TGFβ signaling pathway have been developed, focusing on inhibiting upstream TGFβ and its receptors through the use of neutralizing antibodies, receptor kinase inhibitors, and anti-sense oligonucleotides (Ciardiello et al., 2020; Liu et al., 2021). Although promising results have been obtained with these anti-TGFβ therapies in preclinical *in vitro* studies and mouse models, most clinical trials have failed to reproduce these favorable outcomes (Teixeira et al., 2020; Metropulos et al., 2022). The formidable challenges encountered in the clinical translation of anti-TGFβ signaling therapy not only necessitate the development of novel mechanism-driven strategies but also underscore the imperative to expand the existing repertoire of anti-TGFβ therapeutic options.

Mothers against decapentaplegic homolog 4 (SMAD4) serves as a critical downstream master regulator in the canonical TGFβ signaling pathway (Zhao et al., 2018). It functions as a common SMAD and acts as an adaptor protein, forming protein‒protein interaction (PPI) complexes with receptor-regulated SMADs (R-SMADs), such as SMAD3 (Tang et al., 2021a; Mo et al., 2022). Following the activation of the TGFβ pathway, the SMAD4–SMAD3 PPI complex translocates into the nucleus, binds to SMAD-binding elements (SBEs) containing DNA sequences, and initiates the expression of a wide spectrum of TGFβ target genes. However, SMAD4 has been considered ‘undruggable’ due to its lack of enzymatic activity and its large PPI interface (Ivanov et al., 2013).

**Figure 1:**
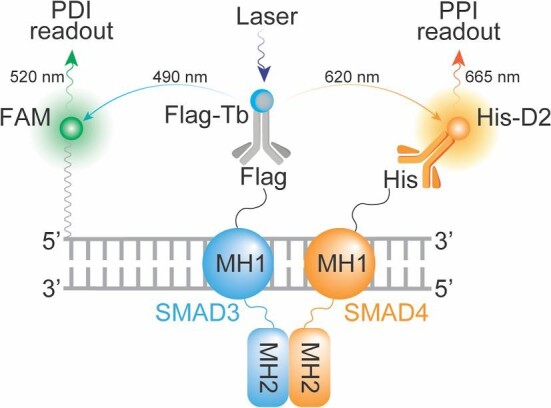
Principle and design of a multiplexed TR-FRET assay. Schematic illustration of the TR-FRET assay with dual-readouts for monitoring the SMAD4–SMAD3 PPI and SMAD3–SBE4 PDI.

The fluorescence resonance energy transfer (FRET) assay is a versatile biosensor technology to study molecular interactions and discover small-molecule modulators (Chu et al., 2011, 2012; Li et al., 2017; Xiong et al., 2018; Tang et al., 2021a, b; Mo et al., 2022). In recent years, terbium (Tb) and other lanthanide chelates, which are long-lived fluorophores (>1 ms), have been used as FRET donors to enable the time-resolved or delayed measurement of the acceptor emission (FRET) signal. The main advantage of the time-delayed measurement is to eliminate the background signal of short-lived fluorescence (ps–ns) from biological samples or screening compounds (Selvin and Hearst, 1994). Therefore, to advance the search for SMAD4 inhibitors and broaden the arsenal of anti-TGFβ signaling therapy, we present the development of a multiplexed time-resolved FRET (TR-FRET) assay capable of simultaneously measuring the SMAD4–SMAD3 PPI and SMAD–SBE protein‒DNA interaction (PDI) (Figure 1). This assay enables the precise monitoring of the dynamics of SMAD4–SMAD3–SBE PPI and PDI at the resolution of single amino acids within a homogeneous cell lysate-based configuration. Furthermore, the assay has been miniaturized and validated for ultrahigh-throughput screening (uHTS) in 1536-well plate format. This optimized and validated uHTS assay will facilitate future large-scale screening campaigns for SMAD4 inhibitors in the quest for novel anti-TGFβ signaling therapy drugs.

## Results

### Design of the multiplexed SMAD4–SMAD3–DNA TR-FRET assay

In the canonical TGFβ signaling pathway, SMAD4 engages in a cooperative interaction with SMAD3 through its Mad homology 2 (MH2) domain, while its MH1 domain interacts with SBE-containing DNA (Figure 1; Stroschein et al., 1999). Thus, to expedite the discovery of SMAD4 inhibitors through high-throughput screening (HTS), the development of a robust and scalable bioassay capable of simultaneously monitoring the cooperative dynamics of SMAD4–SMAD3–DNA PPI and PDI is highly desirable. With this objective in mind, we investigated the feasibility of a cell lysate-based TR-FRET assay (Li et al., 2017; Tang et al., 2021b).

TR-FRET is a well-established bioassay widely utilized for monitoring molecular interactions. In essence, the TR-FRET signal arises from a proximity-based resonance energy transfer (<10 nm) between a long-lived donor fluorophore, such as Tb, and an acceptor fluorophore with a spectrum that overlaps with the donor. Tb exhibits a distinctive fluorescence spectrum with four distinct emission peaks at 490 nm, 546 nm, 583 nm, and 620 nm (Jeyakumar and Katzenellenbogen, 2009). This unique fluorescence emission profile of Tb enables the development of multiplexed TR-FRET assays by employing Tb as a single donor paired with multiple spectrally distinct acceptors. This multiplexing capability allows for the simultaneous monitoring of multiple molecular interactions.

To facilitate the monitoring of the SMAD4–SMAD3–DNA complex, we devised a multiplexed TR-FRET assay comprising the following components: (i) co-expression of His-tagged SMAD4 and Flag-tagged SMAD3 in a cell lysate-based format, (ii) utilization of fluorophore-conjugated anti-fusion-tag antibodies, such as anti-Flag-Tb and anti-His-D2, and (iii) employment of synthesized double-stranded DNA (dsDNA) containing SBEs that are covalently labeled with a FAM fluorophore (Figure 1). This assay configuration enables the simultaneous monitoring of the SMAD4–SMAD3 PPI and SMAD–DNA PDI through the detection of Tb–D2 and Tb–FAM TR-FRET signals, respectively.

### Development of the multiplexed SMAD4–SMAD3–DNA TR-FRET assay

**Figure 2:**
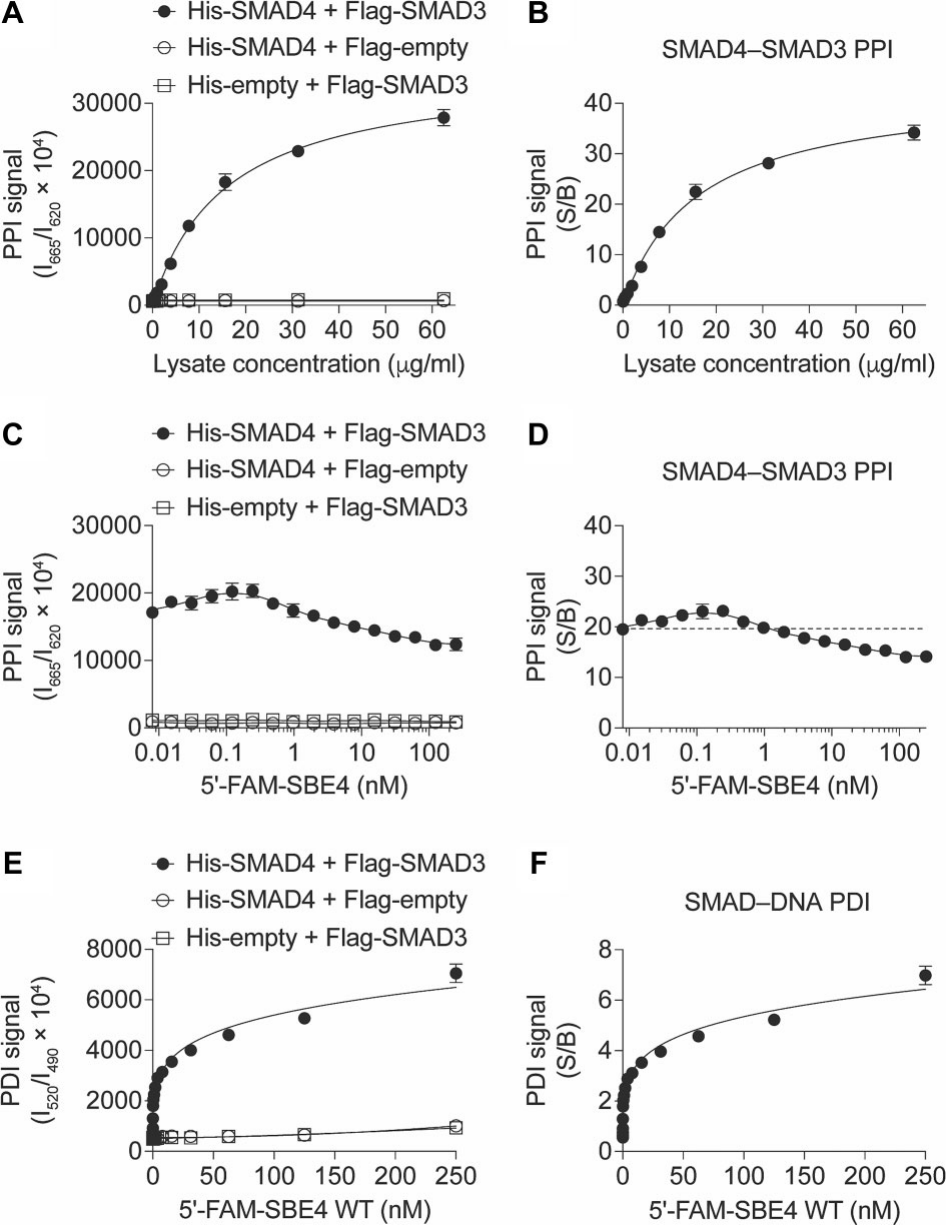
TR-FRET assay development for monitoring the SMAD4–SMAD3–SBE4 complex. (**A** and **B**) Cell lysate dose-dependent curves of the Tb–D2 TR-FRET PPI signal (**A**) and S/B (**B**) from the cell lysates co-expressing His-SMAD4 and Flag-SMAD3 or corresponding controls as indicated. (**C** and **D**) DNA dose-dependent curves of the Tb–D2 TR-FRET PPI signal (**C**) and S/B (**D**) from the lysates co-expressing His-SMAD4 and Flag-SMAD3 or corresponding controls with the titration of 5′-FAM-SBE4 dsDNA as indicated. (**E** and **F**) DNA dose-dependent curves of the Tb–FAM TR-FRET PDI signal (**E**) and S/B (**F**) from the lysates co-expressing His-SMAD4 and Flag-SMAD3 or corresponding controls with the titration of 5′-FAM-SBE4 dsDNA as indicated. The data are expressed as mean ± SD from triplicates from three independent experiments.

To assess the feasibility of the designed assay configuration, we initially optimized the TR-FRET assay using the known SMAD4–SMAD3 PPI as a positive control. According to the assay design, the Tb and D2 fluorophores are brought into close proximity, generating a Tb–D2 TR-FRET signal upon direct interaction between SMAD4 and SMAD3. Consistent with the design, we observed that the cell lysate containing His-tagged SMAD4 and Flag-tagged SMAD3 exhibited significantly higher TR-FRET signals than the corresponding negative controls in a concentration-dependent manner (Figure 2A), with the signal-to-background ratio (S/B) reaching up to 30 (Figure 2B). These results not only confirm the feasibility of employing the lysate-based configuration for monitoring the SMAD4–SMAD3 PPI but also establish the optimal lysate concentration for subsequent multiplexing of the SMAD4–SMAD3 PPI SMAD–SBE4 PDI readouts.

To enable the simultaneous monitoring of PPI and PDI, we selected the cell lysate concentration at the EC_60_ (60% maximal effective concentration) condition of the SMAD4–SMAD3 PPI for further evaluation. This concentration was used to test the feasibility of multiplexing the Tb fluorophore with other fluorophores that have distinct spectra from D2 (ex/em: 620 nm/665 nm). Initially, we examined a synthesized dsDNA oligo, 5′-FAM-SBE4, containing four repeats of the SBE sequence, with 6-carboxyfluorescein (FAM, ex/em: 494 nm/525 nm) conjugated at the 5′-end. As the concentration of 5′-FAM-SBE4 increased, we observed significantly higher PDI signals in the cell lysate containing His-tagged SMAD4 and Flag-tagged SMAD3 compared to the corresponding empty vector controls in a DNA concentration-dependent manner (S/B > 7-fold) (Figure 2E and F). Concurrently, from the same samples, we observed stable SMAD4–SMAD3 PPI signals (S/B > 10-fold) (Figure 2C and D). These results not only demonstrate the feasibility of this multiplexed TR-FRET assay for simultaneous monitoring of the SMAD4–SMAD3 PPI and SMAD–DNA PDI from the same sample but also confirm the cooperative interactions between SMAD4, SMAD3, and the SBE-containing dsDNA (Stroschein et al., 1999).

Moreover, we further characterized our assay for quality control by evaluating batch-to-batch protein expression consistency, detection sensitivity, and lysis buffer compatibility. We observed that His-SMAD4 and Flag-SMAD3 displayed consistent expression across three independent experiments, as evidenced by western blot analysis (Supplementary Figure S1A; see also Figure 2). Conversely, the expression levels of His-SMAD4 and Flag-SMAD3 were below the detection limit in Coomassie blue staining (Supplementary Figure S1B), underscoring the high detection sensitivity of our assay. Additionally, lysates prepared using 1% nonidet P-40 (NP-40) lysis buffer and 0.5% Triton X-100 lysis buffer, both commonly employed in mammalian cell lysis, yielded similar PPI and PDI signals (Supplementary Figure S1C). These findings collectively suggest that our assay is robust for the detection of the SMAD4–SMAD3 complex across various experimental conditions with consistent performance.

### The multiplexed TR-FRET assay is sensitive for the detection of SMAD4–SMAD3–DNA complex dynamics

**Figure 3:**
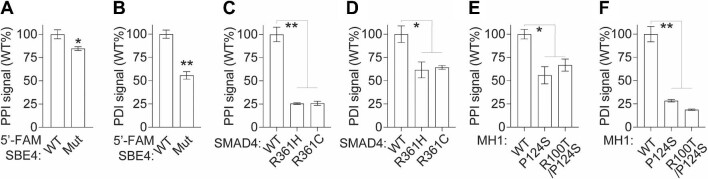
The multiplexed TR-FRET assay allows the detection of SMAD4–SMAD3–DNA complex dynamics. (**A** and **B**) Bar graphs showing the PPI (**A**) and PDI (**B**) signals from cell lysates co-expressing His-SMAD4 and Flag-SMAD3 with 5′-FAM-SBE4 WT or Mut dsDNA. (**C** and **D**) Bar graphs showing the PPI (**C**) and PDI (**D**) signals from cell lysates co-expressing Flag-SMAD3 and His-SMAD4 WT or MH2 domain variants with 5′-FAM-SBE4 WT dsDNA. (**E** and **F**) Bar graphs showing the PPI (**E**) and PDI (**F**) signals from cell lysates expressing SMAD3 and SMAD4 WT or MH1 domain variants with 5′-FAM-SBE4 dsDNA. The data are expressed as mean ± SD from triplicates from three independent experiments. *P < 0.01, **P < 0.001.

To further assess the assay performance in detecting the dynamic interactions of the SMAD4–SMAD3–DNA complex, we examined the differential PPI and PDI signals using SMAD4, SMAD3, and DNA variants that are deficient in complex formation. Initially, we compared the PPI and PDI signals obtained from the cell lysate containing either wild-type (WT) or point-mutated (Mut) 5′-FAM-SBE4 dsDNA. As expected, 5′-FAM-SBE4 Mut dsDNA exhibited a significant reduction in the PDI signal by 45% (*P *< 0.001) and a slight decrease in the PPI signal by 15% (*P *< 0.01) compared to the WT control (Figure 3A and B).

Subsequently, we investigated the PPI and PDI signals using cell lysates expressing SMAD4 R361H and R361C, two naturally occurring cancer-associated hotspot mutations in the MH2 domain that are known to impair the SMAD4–SMAD3 PPI (Alazzouzi et al., 2005; Blackford et al., 2009; Mo et al., 2022). Our analysis revealed that these SMAD3-binding-deficient SMAD4 variants predominantly affected the PPI signal, leading to a substantial reduction of ∼75% (*P *< 0.001) while exhibiting a slight dampening effect on the PDI signal of ∼30% (*P *< 0.01) (Figure 3C and D).

Then, we evaluated the PPI and PDI signals using cell lysates expressing protein variants with point mutations in the MH1 domain and deficient in DNA binding, namely SMAD3 P124S and SMAD4 R100T. Similarly, our findings demonstrated that these DNA-binding-deficient variants resulted in a moderate reduction of ∼25% (*P *< 0.01) in the PPI signal, accompanied by a substantial decrease of ∼75% (*P *< 0.001) in the PDI signal (Figure 3E and F).

Collectively, these findings demonstrate the sensitivity of our multiplexed TR-FRET assay, enabling the study of the dynamic formation of the SMAD4–SMAD3–DNA complex at single-amino acid/nucleotide variant resolution. It also provides further confirmation that this complex is primarily regulated through the cooperative interaction of the SMAD4–SMAD3 PPI mediated by MH2 domains and the SMAD–DNA PDI facilitated by MH1 domains (Stroschein et al., 1999).

### Assay miniaturization into a 1536-well plate to enable uHTS

**Figure 4:**
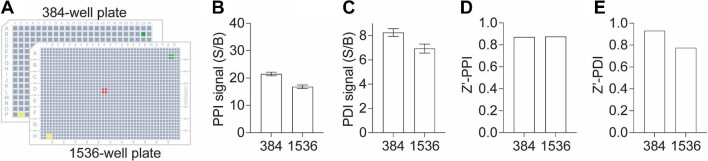
Assay miniaturization for HTS. (**A**) Schematic illustration of assay miniaturization from 384- to 1536-well plate format. (**B** and **C**) Bar graphs showing the S/B ratios of PPI (**B**) and PDI (**C**) signals for the SMAD4–SMAD3–SBE4 WT complex in 384- or 1536-well plate format. (**D** and **E**) Bar graphs showing the Z′ factor of PPI (**D**) and PDI (**E**) signals for the SMAD4–SMAD3–SBE4 WT complex in 384- or 1536-well plate format. The data are expressed as mean ± SD from triplicates from three independent experiments.

To assess the applicability of our multiplexed TR-FRET assay for the discovery of small-molecule modulators, we further miniaturized the assay into a 1536-well plate uHTS format and evaluated its performance for HTS in terms of the S/B ratio and Z-prime factor (Z′) (Figure 4A). The assay demonstrated excellent quality for screening in both 384- and 1536-well plate formats. Robust PPI and PDI signals were achieved in the 1536-well uHTS format with S/B values >15 and ∼6, respectively, along with Z′ values >0.8 and 0.7 (Figure 4B–E). These results underscore the feasibility of utilizing our multiplexed TR-FRET assay for conducting uHTS campaigns to identify small-molecule modulators targeting the SMAD4–SMAD3–DNA complex.

### Pilot screening for the discovery of small-molecule SMAD4–SMAD3–DNA complex inhibitors

**Figure 5:**
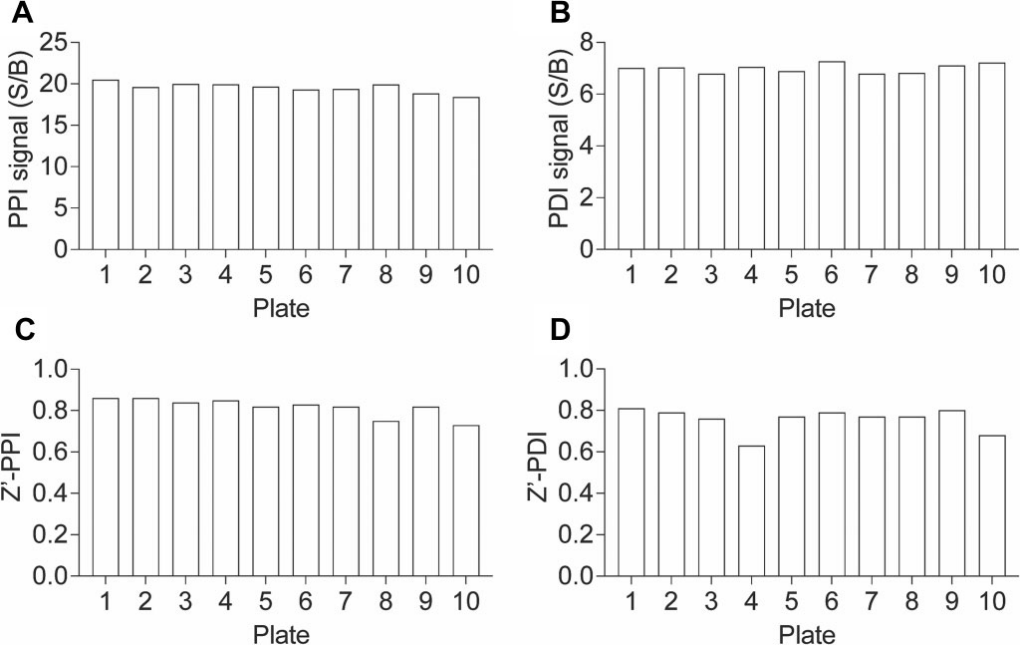
uHTS assay quality control. (**A** and **B**) Bar graphs showing the S/B ratios of PPI (**A**) and PDI (**B**) readouts across 10 plates from the primary screen. The data are expressed as the mean calculated from 16 replicates from the primary screen. (**C** and **D**) Bar graphs showing the Z′ factor of PPI (**C**) and PDI (**D**) readouts across 10 plates from the primary screen.

To validate the suitability of our assay for HTS and small-molecule discovery, we conducted a pilot screening using the Emory Enriched Bioactive Library (EEBL), which comprises 12807 compounds. The primary screening was performed in a 1536-well uHTS format, employing the established conditions described in Figure 4. Each compound was added at a final concentration of 20 μM. For each plate, S/B and Z′ values were calculated for both PPI and PDI signals. Consistently, across 10 1536-well plates, we observed S/B values exceeding 18 and 6 and Z′ values surpassing 0.7 and 0.6 for the PPI and PDI signals, respectively (Figure 5). These consistent and robust results validate the excellent performance of our assay in uHTS applications for small molecule discovery.

**Figure 6:**
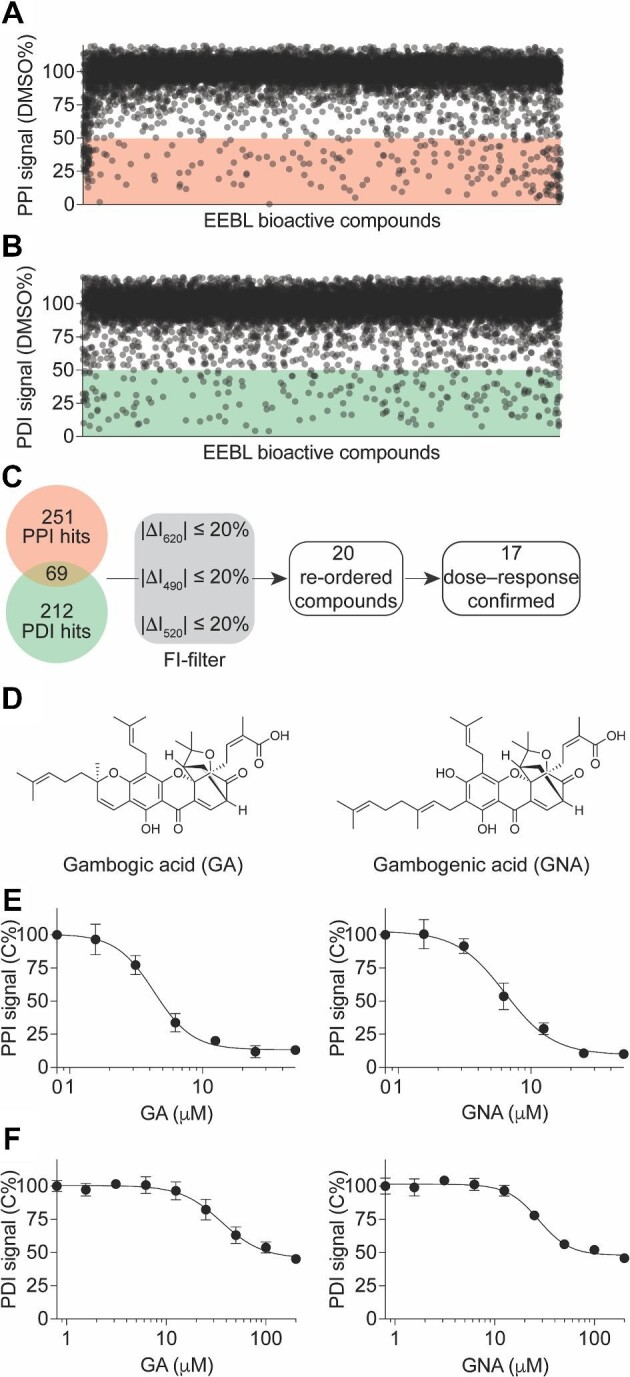
Identification of GA and GNA as SMAD4–SMAD3–SBE4 complex inhibitors from a pilot screening in a 1536-well uHTS format. (**A** and **B**) Scatter plot showing the PPI (**A**) and PDI (**B**) signals induced by compounds from the primary screening. The data are presented as the percentage of the DMSO control from the primary screening. (**C**) Flow chart showing the prioritization of the primary hits. (**D**) Chemical structures of two primary hits, GA and GNA. (**E** and **F**) Dose-dependent curves of GA (left) and GNA (right) in inhibiting the PPI (**E**) and PDI (**F**) signals. The data are presented as mean ± SD from triplicates from one representative experiment.

The screening results are depicted in Figure 6A and B. Using a criterion of ≥50% inhibition compared to the dimethyl sulfoxide (DMSO) control, we identified 251 primary hits from the PPI readout and 212 primary hits from the PDI readout (Figure 6C), corresponding to a hit rate of ∼1.7%–2.0%. Considering the cooperative nature of the SMAD4–SMAD3–DNA complex (Figure 3), we further prioritized 69 hits that showed positive results in both PPI and PDI readouts (Figure 6C). Since the TR-FRET assay relies on fluorescence measurements, we additionally prioritized 20 primary hits by excluding assay interference compounds with fluorescence intensities lower or above 20% in the 490 nm, 520 nm, and 620 nm channels compared to the DMSO control (Figure 6C).

In the TR–FRET dose–response confirmatory assay using cherry-picked and re-ordered compounds, we confirmed 17 out of the 20 primary hits, which exhibited significant and reproducible effects on decreasing the PPI and PDI signals of the SMAD4–SMAD3–DNA complex (Figure 6C). These confirmed hits demonstrated potency in modulating complex formation. The other three primary hits were triaged due to their low potency or lack of consistent effect, indicating that they were not suitable candidates for further investigation.

### Identification of gambogic and gambogenic acid as SMAD4–SMAD3 PPI inhibitors

Among the 17 hits that were confirmed in the dose–response assay, two structurally similar compounds, gambogic acid (GA) and gambogenic acid (GNA) (Figure 6D; Hatami et al., 2020; Hoch et al., 2020), attracted particular attention. These two compounds are polyprenylated xanthone natural products derived from the resin of *Garcinia hanburyi*. In the primary screen, both GA and GNA exhibited robust inhibitory effects on the PPI and PDI signals (Figure 6E and F). Further dose–response confirmation with re-ordered compounds revealed that GA and GNA induced a concentration-dependent decrease in the PPI signal, with half-maximal inhibitory concentrations (IC_50_) of 4.3 ± 0.5 μM and 6.4 ± 1.6 μM, respectively (Figure 6E). Moreover, they also induced a concentration-dependent decrease in the PDI signal, with IC_50_ of 36.0 ± 9.4 μM and 27.6 ± 4.8 μM, respectively (Figure 6F).

**Figure 7:**
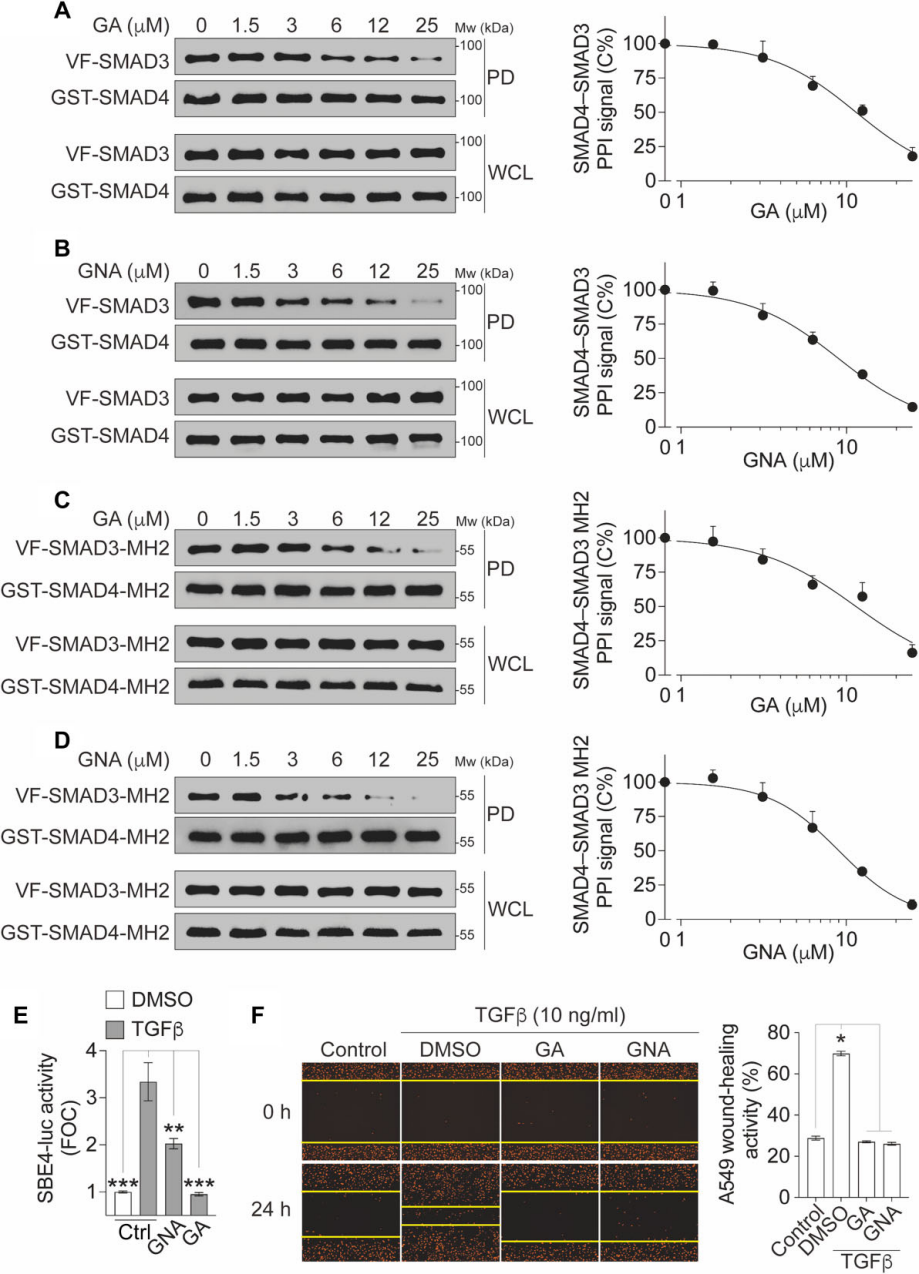
Confirmation of GA and GNA in GST pull-down and cellular functional assays. (**A**–**D**) The inhibition of full-length SMAD4–SMAD3 PPI (**A** and **B**) or SMAD4–SMAD3 MH2 domain PPI (**C** and **D**) by GA (**A** and **C**) or GNA (**B** and **D**). The cell lysates expressing GST-SMAD4 and Venus-flag-tagged SMAD3 (VF-SMAD3) (**A** and **B**) or GST-SMAD4-MH2 and VF-SMAD3-MH2 (**C** and **D**) were treated with compounds as indicated. Left, protein samples from the GST pull-down (PD) and the whole-cell lysates (WCL) were analyzed by western blotting. Right, dose–response curves of the PPI signal were derived from densitometry analysis of the gels. The data are presented as mean ± SEM from three independent experiments. (**E**) GA and GNA inhibit TGFβ-induced SBE4-luc reporter activity. HEK293T cells expressing endogenous SMAD4 and SMAD3 were treated with TGFβ (10 ng/ml) and/or GA or GNA at 5 μM for 18 h. The TGFβ-induced FOC of the luciferase signals is presented as mean ± SD from three independent experiments. **P < 0.01, ***P < 0.001. (**F**) GA and GNA inhibit TGFβ-induced migration of A549 cells labeled with NucLight Red fluorescence protein in the nucleus. After wound scratch, cells were treated with TGFβ (10 ng/ml) and/or GA or GNA at 5 μM for 18 h. Left, representative images showing A549 cell (red) migration toward the wound scratch area. Right, the wound-healing activity is quantified and presented as mean ± SD from three independent experiments. *P < 0.05.

It has been reported that GA and GNA are cysteine-covalent modifiers (Hatami et al., 2020; Hoch et al., 2020). Considering their stronger inhibitory effect on the PPI compared to the PDI in our assays (Figure 6E and F), we hypothesized that GA and GNA might disrupt the SMAD4–SMAD3–DNA complex by targeting the PPI interface. To investigate this hypothesis, we employed a non-fluorescence affinity-based GST pull-down assay. The results revealed that both GA and GNA dose-dependently decreased the level of SMAD3 protein in the pull-down GST-SMAD4 complex, with IC_50_ of ∼11.4 μM and 8.9 μM, respectively (Figure 7A and B). Furthermore, both GA and GNA significantly inhibited the PPI of SMAD4–SMAD3 MH2 domains, which are the primary domains involved in the PPI, in a dose-dependent manner, with IC_50_ of ∼11.4 μM and 9.0 μM, respectively (Figure 7C and D). These findings suggest that GA and GNA primarily disrupt the SMAD4–SMAD3 PPI by interfering with the interactions occurring at the MH2 domains.

To assess the functional impact of GA and GNA on TGFβ signaling, we evaluated the transcriptional activity as a readout for the SMAD4–SMAD3–DNA complex. Specifically, we examined the SBE4-luciferase (SBE4-luc) activity following TGFβ stimulation. Upon TGFβ stimulation, a significant increase in SBE4-luc activity, with a fold-of-change (FOC) >3, was observed (Figure 7E). However, treatment with GA or GNA resulted in a significant reduction in TGFβ-induced SBE4-luc activity (Figure 7E).

In addition, we further assessed the cellular activity of GA and GNA in TGFβ-driven cancer metastasis. We observed that TGFβ treatment significantly enhanced A549 cell migration in wound-healing assays (Figure 7F). However, this TGFβ-induced migration was significantly reduced upon treatment with GA or GNA (Figure 7F). These findings suggest that GA and GNA may attenuate A549 cell migration at least in part by inhibiting TGFβ–SMAD4 signaling. Taken together, the identification of GA and GNA as positive hits suggests the potential druggability of the SMAD4–SMAD3–DNA complex and paves the way for further exploration in small molecule screening campaigns aimed at discovering novel therapeutics targeting the TGFβ signaling pathway.

## Discussion

DNA-binding transcription factors (TFs) hold immense therapeutic potential in cancer treatment (Bushweller, 2019; Henley and Koehler, 2021). However, targeting TFs, other than the nuclear receptor family, poses significant challenges due to their intrinsic disorder and lack of classical binding pockets (Ivanov et al., 2013; Bushweller, 2019; Henley and Koehler, 2021). In this study, we successfully developed a well-designed, optimized, miniaturized, and validated multiplexed TR-FRET assay platform to target the SMAD4–SMAD3–DNA complex. This multiplexed assay has demonstrated exceptional sensitivity in investigating the dynamics of the SMAD4–SMAD3–DNA complex at the resolution of single amino acids. It is readily applicable for large-scale small molecule screening campaigns. Through a pilot chemical screen using this assay, we identified GA and GNA as potential inhibitors of the SMAD4–SMAD3–DNA complex. These compounds not only disrupted the physical PPIs and PDIs within the complex but also effectively inhibited the transcriptional activity induced by TGFβ signaling. These findings underscore the utility of the multiplexed TR-FRET assay for small molecule screening campaigns and provide proof-of-concept evidence supporting the feasibility of directly targeting the previously considered ‘undruggable’ SMAD4–SMAD3–DNA complex. The discovery of small-molecule inhibitors for challenging targets, such as TFs, expands the possibilities for therapeutic interventions in cancer and holds great promise for the development of novel anti-cancer treatments.

SMAD4 acts as an adaptor protein, mediating PPIs, particularly with R-SMADs such as SMAD3, through its MH2 domain (Chacko et al., 2004). This interaction enables the translocation of the SMAD4–SMAD3 complex into the nucleus, where SMADs govern transcription by recognizing DNA sequences containing SBE motifs through their MH1 domain (Shi et al., 1998). The formation of the SMAD4–SMAD3–DNA complex occurs in a highly cooperative manner, playing a crucial role in finely regulated transcriptional processes (Stroschein et al., 1999). This cooperativity has been extensively documented through low-throughput assays, such as the electrophoretic mobility shift assay, utilizing purified proteins (Stroschein et al., 1999). Interestingly, in our cell lysate-based multiplexed TR-FRET assay, we observed that naturally occurring mutations within the MH2 domain of SMAD4 not only disrupt the PPI with SMAD3 (Figure 3C) but also reciprocally impair the complex formation with DNA (Figure 3D). Similarly, mutations in the MH1 domain can affect the PPI (Figure 3E). These findings provide further support for cooperative binding of the SMAD4–SMAD3–DNA complex. The same assay setting could also be applied to study other R-SMADs, such as SMAD2, which can also form complexes with SMAD4 (Chacko et al., 2004). Furthermore, our multiplexed TR-FRET platform, as described, holds the potential to be utilized for studying the dynamics of other TF–DNA complexes in a straightforward and quantitative cell lysate-based high-throughput format.

SMAD4 serves as a crucial downstream master regulator of TGFβ signaling, making SMAD4 inhibitors valuable additions to the anti-TGFβ therapy arsenal. However, the development of SMAD4 inhibitors has been challenging due to the protein's lack of enzymatic activity and its extensive PPI and PDI interfaces. To date, there are no small-molecule inhibitors specifically targeting the SMAD4–SMAD3 interaction. An indole derivative, known as SIS3 (Jinnin et al., 2006), was previously proposed as a potential SMAD3 inhibitor. However, its mode of action involves inhibiting SMAD3 phosphorylation, which indirectly disrupts the SMAD4–SMAD3 PPI. In this study, we present a sensitive and robust multiplexed TR-FRET assay capable of simultaneously monitoring the PPI and PDI signals of the SMAD4–SMAD3–DNA complex in cell lysates. This innovative assay enables us to identify small-molecule inhibitor candidates, such as GA and GNA, which exhibit the potential to directly interfere with the dynamics of the complex.

Polyprenylated xanthone natural products, such as GA and GNA (Hatami et al., 2020; Hoch et al., 2020), have recently garnered attention due to their diverse biological activities, including anti-cancer, anti-inflammatory, antioxidant, and anti-bacterial effects. However, the polypharmacological properties of these compounds are context-dependent and not yet fully understood (Hatami et al., 2020; Hoch et al., 2020). GA and GNA possess electrophilic characteristics that make them prone to cysteine-covalent modification through easy ring opening. Chemoproteomics profiling studies have revealed that GA and its analogs can covalently interact with several protein targets, such as thioredoxins (Pan et al., 2017), transferrin receptor protein 1 (Kasibhatla et al., 2005), and serine palmitoyltransferase (Hoch et al., 2020), in a cysteine thiol-dependent manner. In our study, we found that GA and GNA reduced the PPI signal more substantially than the PDI signal (Figure 6E and F). Additionally, both GA and GNA were observed to disrupt the full-length SMAD4–SMAD3 PPI in GST pull-down assays, which were devoid of DNA oligonucleotides (Figure 7A and B). Moreover, GA and GNA exhibited a dose-dependent disruption of the SMAD4–SMAD3 interaction within their MH2 domains (Figure 7C and D), which mediate PPI but not PDI (Figure 1). These findings collectively suggest that GA and GNA likely disrupt the SMAD4–SMAD3 PPI by targeting their MH2 domains. Further investigations are warranted to elucidate whether GA and GNA modulate the SMAD4–SMAD3–DNA complex by interacting with the cysteine residues within the MH2 domain of the proteins.

The identification of polyprenylated xanthones in this study, including GA and GNA, represents the pioneering class of inhibitors targeting the SMAD4–SMAD3 PPI. The discovery of these compounds holds great promise as a chemical scaffold for the development of potential clinical candidates, particularly as cancer immunotherapy adjuvants for advanced tumors. The effectiveness of the multiplexed TR-FRET uHTS assay provides a proof of concept to support expanded large-scale chemical screening campaigns for the discovery of novel SMAD4–SMAD3 inhibitors.

## Materials and methods

### Cell culture

All cell lines were incubated at 37°C in humidified conditions with 5% CO_2_. Human embryonic kidney 293T cells (HEK293T; ATCC, CRL-3216) were maintained in Dulbecco's modified Eagle's medium (DMEM; Corning, #10-013-CV). A549 NucLight Red cells (Sartorius, Cat# 4491) were maintained in Roswell Park Memorial Institute 1640 Medium (RPMI-1640; #10-040-CV). Cell culture medium was supplemented with 10% fetal bovine serum (FBS; ATLANTA biologicals, #S11550) and 100 units/ml penicillin/streptomycin (Cell Gro, Cat# 30-002-CI).

### Molecular cloning and mutagenesis

The WT SMAD3 (Clone# IOH27044) and SMAD4 (Clone# IOH3638) genes in the pDONR221 plasmid were gifted from Drs Gordon Mill and Yiu Huen Tsang at Oregon Health Science University. The SMAD4 point mutations, including R361H, R361C, and R100T, and the SMAD3 point mutation P124S were introduced using QuikChange Lightning Site-Directed Mutagenesis Kit (Agilent Technologies) with the SMAD4 or SMAD3 pDONR221 plasmid, respectively, as DNA template and the corresponding primers. The SMAD4 and SMAD3 MH2 domain truncation plasmids were generated by polymerase chain reaction using the Platinum™ *Taq* DNA Polymerase kit (Thermo Fisher Scientific) with the SMAD4 or SMAD3 pDONR221 plasmid, respectively, as DNA template and the corresponding primers. Gateway cloning (Invitrogen) was used to generate GST-tagged, Venus-flag-tagged, Flag-tagged, and 6×His-tagged plasmids as previously described (Mo et al., 2016, 2017, 2022; Li et al., 2017; Xiong et al., 2018; Tang et al., 2021b). The vector backbones are pDEST27 vector (Invitrogen) for GST-tag, pDEST26 (Invitrogen) for 6×His tag, pSCM167 for Venus-flag-tag, and pcDNA3.2-V5-dest for Flag-tag constructs. All plasmids generated were confirmed by sequencing. Oligonucleotides used in the study are listed in Table 1.

**Table 1:** Oligonucleotides used in this study.

	**Sequence (5′–3′)**
SMAD4-319-552-F	GGGGACAAGTTTGTACAAAAAAGCAGGCTTCGAAGGAGATAGAACCATGGATATGGCTCCTGAGTATTGGTGTTCCATT
SMAD4-319-552-R	GGGGACCACTTTGTACAAGAAAGCTGGGTTGTCTAAAGGTTGTGGGTCTGCAAT
SMAD3-219-426-F	GGGGACAAGTTTGTACAAAAAAGCAGGCTTCGAAGGAGATAGAACCATGGATATGTTGGACCTGCAGCCAGTTACC
SMAD3-219-426-R	GGGGACCACTTTGTACAAGAAAGCTGGGTTAGACACACTGGAACAGCGG
SMAD4-R361H-F	CTTCTGGAGGAGATCACTTTTGTTTGGGTCAAC
SMAD4-R361H-R	GTTGACCCAAACAAAAGTGATCTCCTCCAGAAG
SMAD4-R361C-F	CCTTCTGGAGGAGATTGCTTTTGTTTGGGTCAA
SMAD4-R361C-R	TTGACCCAAACAAAAGCAATCTCCTCCAGAAGG
SMAD4-R100T-F	TGCCCGTCTCTGGACGTGGCCTGATCTTCA
SMAD4-R100T-R	TGAAGATCAGGCCACGTCCAGAGACGGGCA
SMAD3-P124S-F	AGGTCTGCGTGAATTCCTACCACTACCAGA
SMAD3-P124S-R	TCTGGTAGTGGTAGGAATTCACGCAGACCT
SBE4-FAM	CGCGTAAGAGCTCGGTACGTCTAGACTGCCGTCTAGACTTAGTACGTCTAGACTGCCGTCTAGACTTAGTACCTATCGATAGC/36-FAM/
FAM-SBE4	/56-FAM/CGCGTAAGAGCTCGGTACGTCTAGACTGCCGTCTAGACTTAGTACGTCTAGACTGCCGTCTAGACTTAGTACCTATCGATAGC
SBE4-Mut-FAM	CGCGTAAGAGCTCGGTACGTCTACAGTGCCGTCTACAGTTAGTACGTCTACAGTGCCGTCTACAGTTAGTACCTATCGATAGC/36-FAM/
FAM-SBE4-Mut	/56-FAM/CGCGTAAGAGCTCGGTACGTCTACAGTGCCGTCTACAGTTAGTACGTCTACAGTGCCGTCTACAGTTAGTACCTATCGATAGC
SBE4-RC	GCTATCGATAGGTACTAAGTCTAGACGGCAGTCTAGACGTACTAAGTCTAGACGGCAGTCTAGACGTACCGAGCTCTTACGCG
SBE4-RC-Mut	GCTATCGATAGGTACTAACTGTAGACGGCACTGTAGACGTACTAACTGTAGACGGCACTGTAGACGTACCGAGCTCTTACGCG

### Multiplexed TR-FRET assay

TR-FRET assays were performed using cell lysates from HEK293T cells expressing Flag-tagged SMAD3 and His-tagged SMAD4 WT or mutated proteins. The FRET buffer used throughout the assay contained 20 mM Tris-HCl, pH 7.0, 50 mM NaCl, and 0.01% NP-40. Briefly, HEK293T cells were transiently co-transfected with Flag-tagged SMAD3 WT (1.5 μg/well) or mutated (1.5 μg/well) and His-tagged SMAD4 WT (1.5 μg/well) or mutated (1.5 μg/well) plasmids in 6-well plates. FuGENE (Promega, Cat# E2312) was used as the transfection reagent at a 3:1 (FuGENE/plasmid mass) ratio. After 48 h of transfection, cell lysates were prepared in 200 μl of 1% NP-40 or 0.5% Triton X-100 lysis buffer containing 150 mM NaCl, 10 mM HEPES, pH 7.5, 1% NP-40 (IGEPAL CA-630, Sigma‒Aldrich) or 0.5% Triton X-100 (Sigma‒Aldrich), 5 mM sodium pyrophosphate, 5 mM NaF, 2 mM sodium orthovanadate, 10 mg/L aprotinin, 10 mg/L leupeptin, and 1 mM PMSF.

To determine the optimal cell lysate concentration for HTS, the cell lysate concentration-dependent TR-FRET assay was performed in black 384-well plates (Corning Costar, #3573). Briefly, 15 μl of stock cell lysate was 2-fold serially diluted in FRET buffer and mixed with 15 μl mixture of fluorophore-conjugated antibodies. The total volume for each well was 30 μl containing the cell lysate, anti-FLAG M2-Tb cryptate antibody (Cisbio 61FG2TLF, 1:1000 dilution), and anti-6×His-D2 antibody (Cisbio 61HISDLF, 1:500 dilution). The plate was centrifuged at 500×*g* for 5 min and incubated at 4°C for 30 min. TR-FRET signals for PPI were measured using the BMG Labtech PHERAstar FSX reader with the HTRF optic module (excitation at 337 nm, emission A at 665 nm, emission B at 620 nm, integration start at 50 μs, integration time for 150 μs, and 8 flashes per well). All FRET signals were expressed as a TR-FRET ratio: F665 nm/F620 nm × 10000.

To determine the optimal fluorescein-tagged oligo concentration for HTS, the oligo concentration-dependent TR-FRET assay was performed in black 384-well plates (Corning Costar, #3573). Briefly, 15 μl of stock oligo mixture containing pre-diluted cell lysate (with the desired concentration) and fluorescein-tagged oligos (500 nM) was 2-fold serially diluted in the same concentration of pre-diluted cell lysate and mixed with 15 μl mixture of fluorophore-conjugated antibodies. The total volume for each well was 30 μl containing the fluorescein-tagged oligos, cell lysate, anti-FLAG M2-Tb cryptate antibody (Cisbio 61FG2TLF, 1:1000 dilution), and anti-6×His-D2 antibody (Cisbio 61HISDLF, 1:500 dilution). The plate was centrifuged at 500× *g* for 5 min and incubated at 4°C for 30 min. TR-FRET signals for PDI were measured using the BMG Labtech PHERAstar FSX reader with the HTRF optic module (excitation at 337 nm, emission A at 520 nm, emission B at 490 nm, integration start at 50 μs, integration time for 150 μs, and 8 flashes per well). TR-FRET signals for PPI were measured using the BMG Labtech PHERAstar FSX reader with the HTRF optic module (excitation at 337 nm, emission A at 665 nm, emission B at 620 nm, integration start at 50 μs, integration time for 150 μs, and 8 flashes per well). PDI signals were expressed as a TR-FRET ratio: F520 nm/F490 nm × 10000. PPI signals were expressed as a TR-FRET ratio: F665 nm/F620 nm × 10000.

### uHTS TR-FRET screening for small-molecule PPI inhibitor discovery

uHTS for small-molecule PPI inhibitor discovery was performed using the TR-FRET assay in black 1536-well plates (Corning Costar, #3724) with a total volume of 5 μl in each well. The amount of cell lysate and antibodies were scaled down proportionally from the conditions with the optimal assay window identified from 384-well plates. Briefly, solutions containing cell lysate and antibodies at desired concentrations were dispensed in 1536-well plates (5 μl in each well) using a Multi-Drop Combi Reagent Dispenser (Thermo Scientific). The last column was used as the empty vector background control. Subsequently, the EEBL compounds (100 nl) were added into wells in each plate using Biomek NXP Automated Workstation (Beckman) from a compound stock plate to give a final concentration of 20 μM. The choice of a final compound concentration of 20 μM is a standard practice in biochemical-based HTS. This concentration represents a compromise between compound solubility and achieving a reasonable hit rate. It also ensures that the positive hits exhibit a satisfactory level of potency, with IC_50_ < 20 μM, based on a hit cut-off criterion of >50% inhibition. The final DMSO concentration was 2% (*v*/*v*) in samples with compound treatment. Each sample was tested with a single point. After overnight incubation at 4°C, the FRET signal was measured using the BMG Labtech PHERAstar FSX reader with the HTRF optic module. To evaluate the performance of the assay for HTS, the Z′ factor and S/B ratio were calculated for the TR-FRET titration experiment according to the following equations:


\begin{eqnarray*}
{\mathrm{Z^{\prime}}} = 1-\left( {3 \times {\mathrm{S}}{{\mathrm{D}}}_{{\mathrm{sample}}} + 3 \times {\mathrm{S}}{{\mathrm{D}}}_{{\mathrm{control}}}} \right)/\left( {{\mathrm{FRE}}{{\mathrm{T}}}_{{\mathrm{sample}}}\,\\!-\\!\,{\mathrm{FRE}}{{\mathrm{T}}}_{{\mathrm{control}}}} \right)\end{eqnarray*}

\begin{eqnarray*}
\text{and}\quad {\mathrm{S}}/{\mathrm{B}} = \left( {{\mathrm{FRE}}{{\mathrm{T}}}_{{\mathrm{sample}}}} \right)/\left( {{\mathrm{FRE}}{{\mathrm{T}}}_{{\mathrm{control}}}} \right)\!,\end{eqnarray*}

where SD_sample_ and SD_control_ are standard deviations, and FRET_sample_ and FRET_control_ represent the TR-FRET signals (PPI or PDI) from lysate samples containing oligos, His-SMAD4, and Flag-SMAD3 or containing oligos and empty Flag-vector controls, respectively. The S/B ratio suggests the signal window of the assay, and the Z′ factor reflects the robustness of the assay for HTS. A Z′ factor between 0.5 and 1 indicates a robust assay suitable for HTS. Screening data were analysed using Bioassay software from CambridgeSoft. The effect of compound on PPI and PDI modulation was quantified as the change in TR-FRET signal (ΔTR-FRET) upon compound treatment using the equation 100 × (FRET_compound_—FRET_DMSO_)/FRET_DMSO_, where FRET_compound_ and FRET_DMSO_ are the TR-FRET signals from PPI or PDI in the presence of library compound and DMSO, respectively, with background FRET_vector_ subtracted. A cut-off of ΔTR-FRET ≥ 50% was used to prioritize the positive hits.

### GST pull-down assay

To validate the hits from the pilot screening, we performed the orthogonal GST pull-down assay using cell lysates from HEK293T cells transfected with Venus-flag-SMAD3 and GST-SMAD4 or Venus-flag-SMAD3-MH2 and GST-SMAD4-MH2. After 48 h of transfection, the cells were lysed in 1% NP-40 lysis buffer, incubated with compounds for 16 h with rotation at 4°C, and then incubated with glutathione-conjugated beads (GE 17527901) for 2 h at 4°C. Beads were washed twice with 1% NP-40 lysis buffer, eluted by boiling in sodium dodecyl sulfate (SDS) sample buffer (Bio-Rad), and subjected to western blot analysis.

### Western blotting

Proteins in SDS sample buffer were resolved by 10% SDS polyacrylamide gel electrophoresis and transferred to nitrocellulose filter membranes at 100 V for 2 h at 4°C. After blocking in 5% nonfat dry milk in TBST (20 mM Tris-base, 150 mM NaCl, and 0.05% Tween 20) for 1 h at room temperature, the membranes were blotted with the indicated antibodies, mouse monoclonal anti-FLAG-HRP antibody (Sigma‒Aldrich, # A8592) and rabbit polyclonal anti-GST-HRP antibody (Sigma‒Aldrich, Cat# A7340), at 4°C overnight. The membranes were washed three times with 1× TBST for 15 min. SuperSignal West Pico PLUS Chemiluminescent Substrate (Thermo Fisher Scientific, #34580) and Dura Extended Duration Substrate (Thermo Fisher Scientific, #34076) were used to develop the membranes. Luminescence images were captured using the ChemiDoc^TM^ Touch Imaging System (Bio-Rad).

### SBE luciferase reporter assay

SMAD3/SMAD4 complex transcriptional activity was measured using the SBE-luciferase reporter system. HEK293T cells were used to measure the luciferase activity. Cells were plated in 6-well plates and co-transfected with SBE4-Luc plasmid (1 μg, Addgene, 16495) and pDEST26-Renilla plasmid (0.1 μg) using FuGENE® HD (Promega, #E2312). After 24 h of transfection, the cells were pre-treated with GA (MedChemExpress, #HY-N0087) or GNA (MedChemExpress, #HY-5024) at 5 μM for 6 h followed by TGFβ (10 ng/ml, R&D Systems, #240-B) stimulation for an additional 18 h. Renilla and firefly luciferase activities were measured by an Envision Multilabel plate reader (PerkinElmer) using a Dual-Glo luciferase kit (Promega, Cat# E2920) according to the manufacturer's instructions. The normalized luminescence was calculated as the ratio of the luminescence of firefly luciferase over the luminescence of Renilla luciferase.

### Wound-healing assay

The wound-healing assay was performed using the protocol suggested by the manufacturer (Sartorius). Briefly, A549 NucLight Red cells were seeded in 96-well plates (Greiner, Cat# 655087) at 40000 cells/well in RPMI 1640 media supplemented with 10% FBS and 1% antibiotics. After 24 h, a wound scratch was created in the confluent cell monolayer using a 96-well woundmaker (Sartorius), followed by washing three times with PBS. Then, the medium was replaced with RPMI 1640 medium without FBS. TGFβ (10 ng/ml), GA (5 μM), or GNA (5 μM) was added at the indicated concentrations. After 24 h of treatment, the plates were imaged using the ImageXpress Micro High-Content Imaging System (Molecular Device). The wound-healing activity was quantified by measuring the width (W) of the wound using ImageJ and expressed as (W_0h_—W_24h_)/W_0h_ × 100.

## Supplementary Material

mjad068_Supplemental_File

## Notes

**Author contributions:** conceptualization, X.M., Y.D., and H.F.; assay design and development and chemical screening, W.O., Q.N., M.Q., X.M., and Y.D.; confirmatory assays, W.O., Q.L., and X.M.; data analysis, W.O., Y.D., and X.M.; W.O. and X.M. wrote the initial manuscript, and all were involved in editing.

## References

[bib1] Alazzouzi H., Alhopuro P., Salovaara R. et al. (2005). SMAD4 as a prognostic marker in colorectal cancer. Clin. Cancer Res. 11, 2606–2611. 15814640 10.1158/1078-0432.CCR-04-1458

[bib2] Batlle E., Massague J. (2019). Transforming growth factor-β signaling in immunity and cancer. Immunity 50, 924–940. 30995507 10.1016/j.immuni.2019.03.024 PMC7507121

[bib3] Blackford A., Serrano O.K., Wolfgang C.L. et al. (2009). SMAD4 gene mutations are associated with poor prognosis in pancreatic cancer. Clin. Cancer Res. 15, 4674–4679. 19584151 10.1158/1078-0432.CCR-09-0227 PMC2819274

[bib4] Bushweller J.H. (2019). Targeting transcription factors in cancer—from undruggable to reality. Nat. Rev. Cancer 19, 611–624. 31511663 10.1038/s41568-019-0196-7 PMC8820243

[bib5] Chacko B.M., Qin B.Y., Tiwari A. et al. (2004). Structural basis of heteromeric smad protein assembly in TGF-β signaling. Mol. Cell 15, 813–823. 15350224 10.1016/j.molcel.2004.07.016

[bib6] Chu L., Zhu T., Liu X. et al. (2012). SUV39H1 orchestrates temporal dynamics of centromeric methylation essential for faithful chromosome segregation in mitosis. J. Mol. Cell Biol. 4, 331–340. 22831836 10.1093/jmcb/mjs023 PMC3464394

[bib7] Chu Y., Yao P.Y., Wang W. et al. (2011). Aurora B kinase activation requires survivin priming phosphorylation by PLK1. J. Mol. Cell Biol. 3, 260–267. 21148584 10.1093/jmcb/mjq037 PMC3150119

[bib8] Ciardiello D., Elez E., Tabernero J. et al. (2020). Clinical development of therapies targeting TGFβ: current knowledge and future perspectives. Ann. Oncol. 31, 1336–1349. 32710930 10.1016/j.annonc.2020.07.009

[bib9] Colak S., and Ten Dijke P. (2017). Targeting TGF-β signaling in cancer. Trends Cancer 3, 56–71. 28718426 10.1016/j.trecan.2016.11.008

[bib10] Hahn W.C., Bader J.S., Braun T.P. et al. (2021). An expanded universe of cancer targets. Cell 184, 1142–1155. 33667368 10.1016/j.cell.2021.02.020 PMC8066437

[bib11] Hatami E., Jaggi M., Chauhan S.C. et al. (2020). Gambogic acid: a shining natural compound to nanomedicine for cancer therapeutics. Biochim. Biophys. Acta Rev. Cancer 1874, 188381. 32492470 10.1016/j.bbcan.2020.188381 PMC7484097

[bib12] Henley M.J., Koehler A.N. (2021). Advances in targeting ‘undruggable’ transcription factors with small molecules. Nat. Rev. Drug Discov. 20, 669–688. 34006959 10.1038/s41573-021-00199-0

[bib13] Hoch D.G., Abegg D., Hannich J.T. et al. (2020). Combined omics approach identifies gambogic acid and related xanthones as covalent inhibitors of the serine palmitoyltransferase complex. Cell Chem. Biol. 27, 586–597.e12. 32330443 10.1016/j.chembiol.2020.03.008

[bib14] Hu Y., Hudson W.H., Kissick H.T. et al. (2022). TGF-β regulates the stem-like state of PD-1^+^TCF-1^+^ virus-specific CD8 T cells during chronic infection. J. Exp. Med. 219, e20211574. 35980386 10.1084/jem.20211574 PMC9393409

[bib15] Ivanov A.A., Khuri F.R., Fu H. (2013). Targeting protein‒protein interactions as an anticancer strategy. Trends Pharmacol. Sci. 34, 393–400. 23725674 10.1016/j.tips.2013.04.007 PMC3773978

[bib16] Jeyakumar M., Katzenellenbogen J.A. (2009). A dual-acceptor time-resolved Foster resonance energy transfer assay for simultaneous determination of thyroid hormone regulation of corepressor and coactivator binding to the thyroid hormone receptor: mimicking the cellular context of thyroid hormone action. Anal. Biochem. 386, 73–78. 19111515 10.1016/j.ab.2008.11.039 PMC2701686

[bib17] Jinnin M., Ihn H., Tamaki K. (2006). Characterization of SIS3, a novel specific inhibitor of SMAD3, and its effect on transforming growth factor-β1-induced extracellular matrix expression. Mol. Pharmacol. 69, 597–607. 16288083 10.1124/mol.105.017483

[bib18] Kasibhatla S., Jessen K.A., Maliartchouk S. et al. (2005). A role for transferrin receptor in triggering apoptosis when targeted with gambogic acid. Proc. Natl Acad. Sci. USA 102, 12095–12100. 16103367 10.1073/pnas.0406731102 PMC1189297

[bib19] Li Z., Ivanov A.A., Su R. et al. (2017). The OncoPPi network of cancer-focused protein‒protein interactions to inform biological insights and therapeutic strategies. Nat. Commun. 8, 14356. 28205554 10.1038/ncomms14356 PMC5316855

[bib20] Liu S., Ren J., Ten Dijke P. (2021). Targeting TGFβ signal transduction for cancer therapy. Signal Transduct. Target. Ther. 6, 8. 33414388 10.1038/s41392-020-00436-9 PMC7791126

[bib21] Mariathasan S., Turley S.J., Nickles D. et al. (2018). TGFβ attenuates tumor response to PD-L1 blockade by contributing to exclusion of T cells. Nature 554, 544–548. 29443960 10.1038/nature25501 PMC6028240

[bib22] Marie J.C., Letterio J.J., Gavin M. et al. (2005). TGF-β1 maintains suppressor function and Foxp3 expression in CD4^+^CD25^+^ regulatory T cells. J. Exp. Med. 201, 1061–1067. 15809351 10.1084/jem.20042276 PMC2213134

[bib23] Metropulos A.E., Munshi H.G., Principe D.R. (2022). The difficulty in translating the preclinical success of combined TGFβ and immune checkpoint inhibition to clinical trial. EBioMedicine 86, 104380. 36455409 10.1016/j.ebiom.2022.104380 PMC9706619

[bib24] Mo X., Niu Q., Ivanov A.A. et al. (2022). Systematic discovery of mutation-directed neo-protein‒protein interactions in cancer. Cell 185, 1974–1985.e12. 35512704 10.1016/j.cell.2022.04.014 PMC9597701

[bib25] Mo X., Qi Q., Ivanov A.A. et al. (2017). AKT1, LKB1, and YAP1 revealed as MYC interactors with NanoLuc-based protein-fragment complementation assay. Mol. Pharmacol. 91, 339–347. 28087810 10.1124/mol.116.107623 PMC5363710

[bib26] Mo X.L., Luo Y., Ivanov A.A. et al. (2016). Enabling systematic interrogation of protein‒protein interactions in live cells with a versatile ultra-high-throughput biosensor platform. J. Mol. Cell Biol. 8, 271–281. 26578655 10.1093/jmcb/mjv064 PMC4937889

[bib27] Pan H., Jansson K.H., Beshiri M.L. et al. (2017). Gambogic acid inhibits thioredoxin activity and induces ROS-mediated cell death in castration-resistant prostate cancer. Oncotarget 8, 77181–77194. 29100379 10.18632/oncotarget.20424 PMC5652772

[bib28] Ribas A., Wolchok J.D. (2018). Cancer immunotherapy using checkpoint blockade. Science 359, 1350–1355. 29567705 10.1126/science.aar4060 PMC7391259

[bib29] Selvin P.R., Hearst J.E. (1994). Luminescence energy transfer using a terbium chelate: Improvements on fluorescence energy transfer. Proc. Natl Acad. Sci. USA 91, 10024–10028. 7937831 10.1073/pnas.91.21.10024 PMC44950

[bib30] Shi Y., Wang Y.F., Jayaraman L. et al. (1998). Crystal structure of a Smad MH1 domain bound to DNA: insights on DNA binding in TGF-β signaling. Cell 94, 585–594. 9741623 10.1016/s0092-8674(00)81600-1

[bib31] Stroschein S.L., Wang W., Luo K. (1999). Cooperative binding of SMAD proteins to two adjacent DNA elements in the plasminogen activator inhibitor-1 promoter mediates transforming growth factor β-induced smad-dependent transcriptional activation. J. Biol. Chem. 274, 9431–9441. 10092624 10.1074/jbc.274.14.9431

[bib32] Tang C., Mo X., Niu Q. et al. (2021a). Hypomorph mutation-directed small-molecule protein‒protein interaction inducers to restore mutant SMAD4-suppressed TGF-β signaling. Cell Chem. Biol. 28, 636–647.e5. 33326750 10.1016/j.chembiol.2020.11.010 PMC10053325

[bib33] Tang C., Niu Q., Cicka D. et al. (2021b). A time-resolved fluorescence resonance energy transfer screening assay for discovery of protein‒protein interaction modulators. STAR Protoc. 2, 100804. 34527960 10.1016/j.xpro.2021.100804 PMC8433285

[bib34] Teixeira A.F., Ten Dijke P., Zhu H.J. (2020). On-target anti-TGF-β therapies are not succeeding in clinical cancer treatments: what are remaining challenges? Front. Cell Dev. Biol. 8, 605. 32733895 10.3389/fcell.2020.00605 PMC7360684

[bib35] Wei S.C., Duffy C.R., Allison J.P. (2018). Fundamental mechanisms of immune checkpoint blockade therapy. Cancer Discov. 8, 1069–1086. 30115704 10.1158/2159-8290.CD-18-0367

[bib36] Xiong J., Pecchi V.G., Qui M. et al. (2018). Development of a time-resolved fluorescence resonance energy transfer ultrahigh-throughput screening assay for targeting the NSD3 and MYC interaction. Assay Drug Dev. Technol. 16, 96–106. 29634317 10.1089/adt.2017.835 PMC5865254

[bib37] Zhao M., Mishra L., Deng C.X. (2018). The role of TGF-β/SMAD4 signaling in cancer. Int. J. Biol. Sci. 14, 111–123. 29483830 10.7150/ijbs.23230 PMC5821033

