# Supplementary material for: A multiplexed time-resolved fluorescence resonance energy transfer ultrahigh-throughput screening assay for targeting the SMAD4–SMAD3–DNA complex
Source: J Mol Cell Biol. 2023 Nov 15;15(11):mjad068. doi: 10.1093/jmcb/mjad068 (PMC11063955; doi:10.1093/jmcb/mjad068)
Supplement: mjad068_Supplemental_File [file mjad068_supplemental_file.pdf]

Supplementary material

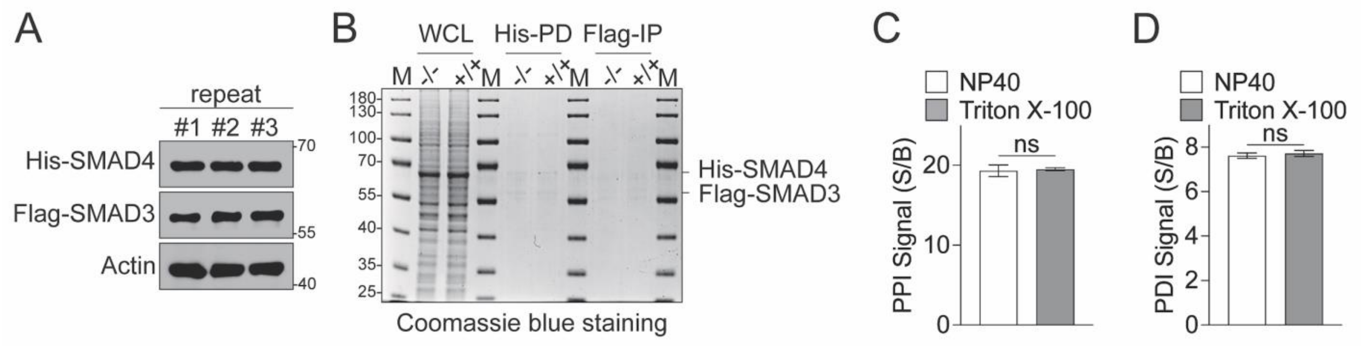

Supplementary Figure S1. Additional assay quality control data. (Related to Fig. 2)

(**A**) Western blot showing consistent expression of His-SMAD4 and Flag-SMAD3 across three independent experiments. Cell lysate from HEK293T cells expressing His-SMAD4 and Flag-SMAD3 were analyzed using SDS-PAGE and blotted with His-, Flag-, and actin-antibodies as indicated.

(**B**) Representative coomassie blue gel showing the expression of His-SMAD4 and Flag-SMAD3. Cell lysates were prepared from HEK293T cells co-transfected with His-SMAD4 and Flag-SMAD3 (+/+) or with His- and Flag-empty control vectors (–/–). WCL: whole cell lysate samples; His-PD: His-tag pulldown samples using Ni-NTA beads; Flag-IP: Flag-tag immunoprecipitation samples using Flag-antibody coated agarose beads; M: protein markers.

(**C** and **D**) Bar graphs showing the PPI (**C**) and PDI (**D**) signals from lysates prepared using 1% NP-40 or 0.5% Triton X-100 lysis buffer. The data are presented as mean ± SD from three independent experiments. ns, $P > 0.1$.
